# Use of Allopurinol to Mitigate 6-Mercaptopurine Associated Gastrointestinal Toxicity in Acute Lymphoblastic Leukemia

**DOI:** 10.3389/fonc.2020.01129

**Published:** 2020-07-16

**Authors:** Shannon E. Conneely, Stacy L. Cooper, Rachel E. Rau

**Affiliations:** ^1^Department of Pediatric Hematology and Oncology, Baylor College of Medicine, Texas Children's Hospital, Houston, TX, United States; ^2^Department of Oncology, Johns Hopkins University School of Medicine, Baltimore, MD, United States

**Keywords:** acute lymphoblastic leukemia (ALL), allopurinol, 6-mercaptopurine, hepatotoxicity, gastrointestinal, hypoglycemia, purine metabolism, 6-methylmercaptopurine

## Abstract

An essential component of acute lymphoblastic leukemia (ALL) therapy is the prolonged maintenance phase with daily 6-mercaptopurine (6-MP) as the cornerstone. While 6-MP is generally well-tolerated, some patients suffer from significant side effects such as gastrointestinal (GI) toxicity, including hepatitis, hypoglycemia, nausea, and pancreatitis, which can substantially limit the tolerated dose of 6-MP. These toxicities are thought to result from skewed metabolism of 6-MP leading to an accumulation of the 6-methylmercaptopurine (6-MMP) metabolite. Here, we describe current knowledge behind the use of allopurinol to modify 6-MP metabolism and improve tolerance to therapy. This method has been successfully used in adults with inflammatory bowel disease refractory to purine therapy and has been modified for use in children with GI toxicities related to 6-MP in maintenance therapy for ALL. Use of allopurinol for 6-MP related toxicities should be reserved for patients in which an alternative cause of signs or symptoms has been excluded and for whom non-pharmacologic measures have failed. When allopurinol is used, simultaneous dose reduction of 6-MP is required to avoid severe myelosuppression and related side effects, though overall combination therapy appears to be well-tolerated and effective when instituted appropriately.

## Introduction

Acute lymphoblastic leukemia (ALL) is the most common cancer in children and includes a prolonged maintenance phase of therapy which has significantly decreased the risk of relapse (1, 2). 6-mercaptopurine (6-MP) is an essential component of maintenance therapy, with daily dosing for up to 2.5 years in combination with other antineoplastic agents. 6-MP is well-tolerated by a majority of patients, yet some children experience significant side effects which can result in substantial pauses in therapy or dose reductions that may affect long-term outcomes and influence quality of life (3). 6-MP is a purine analog which must be metabolized to its active form, 6-thioguanine nucleotide (6-TGN) to achieve desired antineoplastic effects (4). An alternative metabolic pathway involves methylation of 6-MP to 6-methylmercaptopurine (6-MMP) which is believed to be primarily responsible for gastrointestinal (GI) side effects such as refractory nausea, pancreatitis, and hepatotoxicity including elevated liver enzymes and/or hypoglycemia when inappropriately elevated (5, 6). Striking a balance in 6-MP metabolism such that 6-TGN reaches appropriate therapeutic thresholds while minimizing hepatotoxic effects of 6-MMP has been challenging for a select number of pediatric patients.

Allopurinol alters the metabolism of purine analogs, leading to decreased 6-MMP and increased 6-TGN levels, though the mechanism of action is not fully understood (7). It was first used in patients with inflammatory bowel disease (IBD) refractory to 6-MP therapy alone, and concomitant use of 6-MP and allopurinol in this population increases the rate of steroid-free remissions (8). Similar methods have since been employed in ALL patients who experience 6-MP dose-limiting hepatotoxicity or other 6-MP associated side effects, with the goal of reducing 6-MMP levels and associated toxicity while maintaining therapeutic benefit. However, addition of allopurinol may introduce undesired side effects such as GI upset, dermatologic conditions, neutropenia, and increased infection risk (8). Here, we review the use of allopurinol for 6-MP associated GI toxicity in the pediatric ALL population, including recommendations for when and how to institute therapy.

## Purine Metabolism

**Figure 1:**
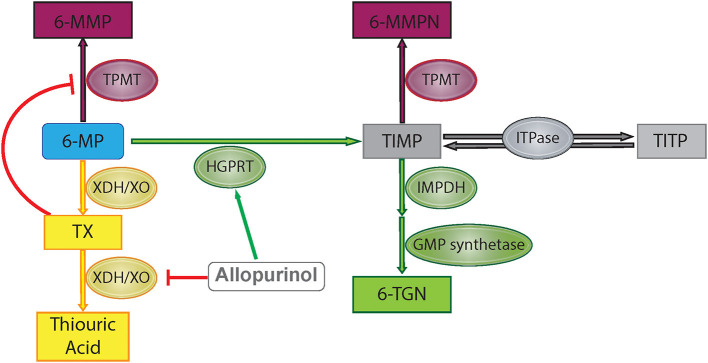
6-Mercaptopurine metabolism with proposed allopurinol mechanisms of action. Green represents HGPRT pathway, yellow represents XO pathway, and maroon represents methylation pathway. 6-MP, 6-mercaptopurine; 6-MMP, 6-methylmercaptopurine; 6-MMPN, 6-methylmercaptopurine nucleotides; TIMP, thio-inosine monophosphate; TITP, thio-inosine triphosphate; TX, thioxanthine; 6-TGN, 6-thioguanine nucleotide; TPMT, thiopurine methyltransferase; HGPRT, hypoxanthine-guanine phosphoribosyltransferase; XDH/XO, xanthine dehydrogenase/xanthine oxidase; IMPDH, inosine monosphosphate dehydrogenase; GMP, guanosine monophosphate; ITPase, inosine triphosphatase pyrophosphatase.

6-MP is a prodrug which is metabolized through several different pathways to reach its active and inactive forms (Figure 1). First, hypoxanthine-guanine phosphoribosyl transferase (HGPRT) enzymatically converts 6-MP to thio-inosine monophosphate (TIMP) which can then be converted to active 6-TGN through a series of additional steps involving inosine monosphosphate dehydrogenase and guanosine monophosphate synthetase (7). Alternatively, 6-MP or the TIMP intermediate can undergo methylation by thiopurine methyltransferase (TPMT), producing the inactive 6-MMP responsible for hepatotoxicity (9). A third pathway involves degradation of 6-MP to thiouric acid by xanthine oxidase and xanthine dehydrogenase. Thiouric acid is inactive and not considered to significantly contribute to tolerance to 6-MP therapy.

Genetic variation in the activity of enzymes involved in the purine salvage pathway is known to alter response to 6-MP and other purine analogs. Up to 10% of patients have a genetic variant leading to decreased activity of TPMT which preferentially shifts metabolism toward 6-TGN production (10). More recently, missense variants in the gene *NUDT15* have also been associated with 6-MP intolerance, as NUDT15 is thought to phosphorylate active 6-MP metabolites to an inactive form, thereby leading to an accumulation of active metabolites when NUDT15 activity is decreased (11). Patients with decreased TPMT or NUDT15 activity can therefore experience severe myelosuppression caused by excessive levels of active 6-MP metabolites. Routine screening for *TPMT* and *NUDT15* variants prior to 6-MP initiation is now recommended by many childhood cancer consortia, with recommendations for pre-emptive reduced dose 6-MP for patients found to have genetic variants associated with severely reduced 6-MP metabolism (12). Genetic variants leading to hypermethylation and accumulation of 6-MMP have not been extensively described.

Allopurinol is a purine analog that inhibits xanthine oxidase, the enzyme responsible for production of uric acid through oxidation of hypoxanthine and xanthine (13). Allopurinol therefore decreases uric acid formation and is used to treat conditions caused by excess uric acid such as gout and renal stones. It is also used in the management of tumor lysis syndrome prior to or after initiation of chemotherapy when malignant cells rapidly lyse and release their intracellular contents, leading to hyperuricemia, and subsequent renal dysfunction secondary to the accumulation of urate crystals (13). Allopurinol use in this setting inhibits uric acid formation, thereby reducing the risk of renal failure.

Allopurinol has also been shown to alter purine metabolism such that production of the active metabolite of 6-MP, 6-TGN, is favored over production of the methylated metabolite, 6-MMP (Figure 1). As xanthine oxidase is involved in the metabolism of 6-MP to thiouric acid, inhibition via allopurinol reduces 6-MP inactivation along this pathway, but this does not explain changes in the relative production of 6-TGN/6-MMP. One proposed mechanism for alterations in 6-MMP and 6-TGN production is that allopurinol indirectly inhibits TPMT activity via inhibition of xanthine dehydrogenase, leading to increased levels of an intermediate compound, thioxanthine, which directly inhibits TPMT (14). Other studies have shown that allopurinol increases HGPRT activity, accelerating the first step in conversion of 6-MP to 6-TGN (15). Concurrent use of 6-MP with allopurinol has been exploited in other diseases treated with purine analogs in order to maximize therapeutic effects without increasing toxicity, with initial studies completed in patients treated with purine analogs for IBD (8). Recently this strategy has been used in children on 6-MP therapy for the treatment of ALL.

## Clinical Use of Allopurinol to Mitigate 6-MP Toxicity during All Maintenance

6-MP remains an essential part of ALL therapy most prominently as continuous daily dosing during maintenance therapy. While a majority of patients experience no serious adverse effects, those that develop GI-related toxicities may present a unique therapeutic challenge. Potential GI side effects include elevated liver enzymes, hyperbilirubinemia, pancreatitis, anorexia, hypoglycemia, vomiting, or abdominal pain. These toxicities vary from mild lab abnormalities and symptoms to those that severely affect quality of life or risk long-term organ damage. When severe GI toxicity occurs as a result of 6-MP, treatment protocols currently recommend holding the drug until resolution of symptoms, sometimes with subsequent dose reduction to minimize potential side effects. While this is often effective at relieving toxicity, the concomitant decrease in 6-TGN may compromise the antineoplastic effects (16, 17). In some cases, 6-MP may be discontinued altogether, placing the patient at higher risk of relapse (3). Concurrent use of allopurinol and 6-MP may provide an opportunity to alleviate toxicity while potentially maintaining the therapeutic benefit of 6-MP.

Consideration should initially be given to whether symptoms are a direct result of 6-MP toxicity or a different etiology, as numerous alternative diagnoses exist. For example, elevated liver enzymes may be a result of viral or fungal infection or medications such as fluconazole, vincristine, and methotrexate. Hypoglycemia may be caused by adrenal insufficiency or other endocrinologic disorders. As many patients enduring chemotherapy also require additional supportive medications, polypharmacy may also contribute to symptoms which mimic 6-MP toxicities. Ultimately, in the absence of other identified etiologies, thiopurine metabolite testing may be useful in assessing the risk for 6-MP associated hepatotoxicity. Early studies in IBD demonstrate that a 6-MMP level above 5,700 pmol/8 × 10^8^ RBC is associated with an increased risk of hepatotoxicity (7). A small study in children with ALL demonstrated increased risk of hepatotoxicity with 6-MMP levels above 5,000 pmol/8 × 10^8^ RBC, a lower threshold than that reported in IBD (9). Current studies in IBD and other diseases use the 6-MMP:6-TGN ratio as an alternative measure of skewed metabolism with a ratio > 20 considered significant, as this has been associated with therapeutic inefficiency in IBD even in the absence of other side effects (7, 8, 18). Of note, the dose of 6-MP required in IBD is lower than that used in ALL maintenance therapy and therapeutic goals differ. While large-scale studies assessing hepatotoxic 6-MP metabolites in pediatric ALL patients have not been reported to date, these values have been applied in some of the case reports of allopurinol use for pediatric ALL appearing in the literature (19). Future studies aimed at validating metabolite levels to guide the management of 6-MP associated GI toxicity are ongoing (NCT03022747; NCT02046694).

6-MP associated GI toxicity has traditionally been managed by modification of 6-MP administration. Historically, 6-MP has been given in the evenings without food based on studies suggesting improved event-free survival for ALL patients when 6-MP is taken in the evening compared to the morning (20, 21). However, more recent studies challenge these claims with no notable change in relapse risk or 6-TGN levels based on administration habits related to timing or co-administration with food, as long as 95% or more of the daily doses are taken (22, 23). Twice daily administration has also been shown to maintain 6-TGN levels similar to daily dosing while reducing 6-MMP levels so long as the total daily dose is maintained (24). Alternative dosing schedules may therefore provide symptomatic relief for some, though the benefit is likely dependent on the exact toxicity experienced by each patient. Thus far, these methods have only been reported in patients experiencing hypoglycemia. Two cases demonstrated complete reversal of symptoms with changes in timing of 6-MP administration plus a carbohydrate-enriched diet, whereas these methods failed to resolve hypoglycemic episodes in two other reports (25–28). A fifth case required 6-MP dose reduction in addition to morning administration to achieve the desired response (29). Alternative dosing regimens may therefore provide a simple solution for select patients without compromising outcomes.

In a proportion of ALL patients with confirmed 6-MP associated GI toxicity not responding to alternate dosing strategies, combined allopurinol and 6-MP has been tried with some success, though data is limited by small numbers and potential underreporting of negative results. Case reports and case series support the combined use of allopurinol and 6-MP for various types of toxicities and has even been reported in patients with elevated 6-MMP levels in the absence of GI toxicity (Table 1). One case report included two patients with 6-MP associated pancreatitis in which both were successfully treated with combination therapy without recurrence of pancreatitis (31). Both patients had previously developed pancreatitis following asparaginase while simultaneously taking 6-MP in pre-maintenance phases, perhaps increasing the likelihood of recurrence with 6-MP alone. Two case reports including four total patients have also been published using combination therapy for 6-MP associated hypoglycemia (27, 28). All four patients experienced complete resolution of symptoms along with lower 6-MMP levels and lower 6-MMP:6-TGN ratio. The most comprehensive data available for ALL patients thus far is from a single institution retrospective review which included 19 patients treated with combination therapy (19). In this review, patients were treated with allopurinol if they had skewed 6-MP metabolism as evidenced by a 6-MMP:6-TGN ratio > 20 in addition to either Grade 2 or higher hepatotoxicity or persistent leukocytosis requiring multiple dose escalations with failure to achieve desired degree of neutropenia. Eighteen out of 19 patients achieved the desired response to combination therapy. Alanine aminotransferase levels fell below Grade 2 toxicity at a median of 22 days following allopurinol initiation. 6-MMP levels were slower to respond, with 6-MMP falling below 5,700 pmol/8 × 10^8^ RBC at a median of 60 days after the start of allopurinol, though changes in 6-MMP:6-TGN ratio are not reported. It is important to note that this was a retrospective review, and thus follow-up testing frequency was not standardized amongst patients. Regardless, these studies indicate a possible role for allopurinol as a tool to mitigate 6-MP associated GI toxicities in ALL.

**Table 1:** Summary of literature on combined allopurinol and 6-mercaptopurine therapy in ALL.

**Publication**	**No. treated**	**No. responding (%)**	**Indications for allopurinol**	**Allopurinol dose**	**Mean 6-MP dose reduction**
Stuckert et al. (19)	19	18 (95)	Skewed 6-MP metabolites plus elevated ANC or hepatotoxicity	50 mg/m^2^/day	54%
Zhang et al. (28)	2	2 (100)	Hypoglycemia	50 mg daily	74.5%
Miller et al. (27)	2	2 (100)	Hypoglycemia	50 mg/m^2^ or 50 mg daily	63%
Giamanco et al. (30)	2	2 (100)	Hepatotoxicity	4 mg/kg/day	48%
Zerra et al. (31)	2	2 (100)	Pancreatitis	50 mg twice daily	66%
Brackett et al. (32)	3	3 (100)	Hyperbilirubinemia Elevated 6-MMP	50 mg/m^2^/day	52%

*Composite data from six series including reason for allopurinol therapy, allopurinol and 6-MP doses, and responses achieved*.

Since allopurinol skews the metabolism of 6-MP toward 6-TGN production with subsequent rise in 6-TGN levels, toxicity secondary to elevated 6-TGN levels is possible when the two are used in combination. A report of an adult case where 6-MP and allopurinol were prescribed independently for different disorders resulted in profound neutropenia and infection (33). While the exact dose in pediatrics is not standardized, 6-MP doses have generally been reduced by 50–75% when allopurinol is initiated (19, 27, 30, 32). IBD studies err on the lower side with 75% dose reduction followed by dose escalation until therapeutic response or dose-limiting side effects occur (8, 34, 35). In the single-institution retrospective study of 19 patients, most had their 6-MP dose reduced by 50% at allopurinol initiation (19). Eighty-nine percent of patients underwent subsequent dose escalations due to elevated neutrophil counts, though only 47% of these patients maintained this higher dose whereas 53% later required dose reduction due to recurrent neutropenia. Allopurinol dosing has also not been standardized, with some reports using a fixed dose such a 50 mg once or twice daily, whereas others use 50 mg/m^2^/day (19, 27, 31, 32). A majority of case reports maintain the same dose of allopurinol throughout therapy for each patient, though in some cases allopurinol is also titrated to the desired response. Ongoing clinical trials in pediatric ALL patients will ultimately help define appropriate dosing in this population.

The use of allopurinol with 6-MP has generally been safe when instituted appropriately with continued monitoring. In IBD where use of allopurinol is reserved for patients refractory to purine therapy and not specifically related to 6-MP associated toxicities, infection is the most common side effect, though this does not occur exclusively in the setting of neutropenia (8). This is followed by GI upset and dermatologic conditions. In ALL, the most common side effects were fever and neutropenia or other cytopenias, with nearly half of patients requiring subsequent holds in 6-MP therapy. Of course, these side effects can also be seen with 6-MP use alone (19, 27, 32). A distinct difference between IBD and ALL use of allopurinol is that neutropenia is a desired effect and target of ALL therapy, whereas this is not the case for IBD, and thus IBD patients may achieve desired therapeutic responses without as much risk for neutropenia.

One issue that has not been addressed in any of these reports is whether or not relapse risk is affected by use of allopurinol during maintenance therapy. Based on the limited data available from case reports, only one patient in any of the available literature has relapsed at the time of publication (19). Generalized conclusions are inherently limited by the small numbers of reported patients with heterogenous diagnoses and differing parent chemotherapy regimens. Additionally, there is limited long-term follow up of the treated patients. The two ongoing clinical trials specifically investigating combination therapy in children with ALL are not designed to investigate long term outcomes, however recommendations for use of allopurinol during maintenance therapy have been incorporated into the recent trials for B-lymphoblastic leukemia and may provide some insight into allopurinol's influence on relapse risk. Studies assessing the relationship between 6-TGN levels and relapse risk in leukemia have previously shown that patients with 6-TGN below the median, roughly 280 pmol/8 × 10^8^ RBC, have an independently increased risk of relapse compared to those with 6-TGN levels above the median (16, 17). Therefore, one could hypothesize that use of allopurinol with resultant increase in 6-TGN levels may reduce relapse risk in this unique population.

## Discussion

6-MP is a critical component of therapy for children with ALL, particularly during the prolonged maintenance phase which may last up to 2.5 years (1, 2). Hepatotoxicity and other GI side effects due to 6-MP, while uncommon, may lead to pauses in chemotherapy, reduced 6-MP dosing, and challenging lifestyle changes that negatively affect both a child's quality of life and potentially increase their risk for relapse. The use of allopurinol to alter 6-MP metabolism has shown benefit in IBD to improve responses to therapy, and similar concepts are now being applied to ALL patients experiencing significant 6-MP side effects related to elevated 6-MMP metabolites. In some cases, skewed 6-MP metabolism may present with inadequate myelosuppression leading to dose escalation of 6-MP which precedes GI toxicity, thus suggesting that earlier institution of allopurinol therapy may also be of interest. Current data is limited but does demonstrate potential benefit in the pediatric ALL population.

**Figure 2:**
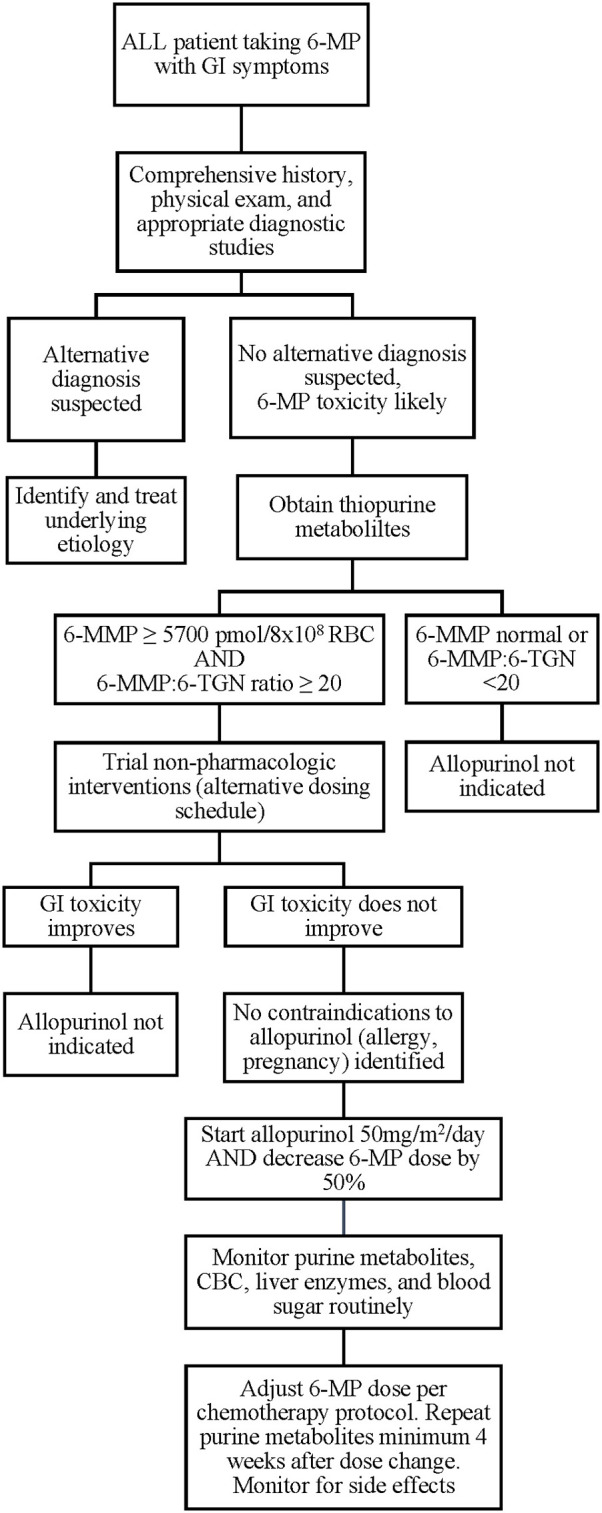
Proposed algorithm for evaluation and implementation of allopurinol for 6-MP associated GI toxicities. 6-MP, 6-mercaptopurine; 6-MMP, 6-methylmercaptopurine.

While the ALL patients reported thus far have experienced no severe adverse effects of combination 6-MP and allopurinol therapy and many were able to achieve the desired changes in 6-MMP and 6-TGN levels with symptomatic improvement, individual patient responses may vary and there is currently no way to predict which patients will respond favorably. In addition, contraindications to allopurinol should be considered, including pregnancy, drug allergy, and drug-drug interactions. If available, allopurinol therapy would best be administered in the context of a clinical trial. Where a clinical trial is not available, we believe initial allopurinol dosing of 50 mg/m^2^/day with 50% dose reduction in 6-MP is a reasonable starting dose for combination therapy after alternative etiologies have been excluded and conservative measures have failed, based on current literature (Figure 2). These should serve only as general suggestions, and providers must carefully assess each clinical situation and act on an individual basis. Following implementation of combination therapy, close monitoring for recurrence of side effects, occurrence of cytopenias, and ongoing monitoring of 6-MMP and 6-TGN levels should be continued to ensure that the desired effect is both attained and maintained. The appropriate dose of both allopurinol and 6-MP should be titrated based on clinical and laboratory response. In addition, current thiopurine metabolite targets are based on a limited number of patients and use of the thresholds described here may fail to identify all patients who might benefit from combination therapy. As described above, optimal dose regimen requires titration based on patient response, and some patients may fail to have resolution of symptoms despite reaching current target 6-TGN and 6-MMP levels. In addition, polypharmacy is a risk of added medication and may affect metabolism and tolerance of other drugs, as well as increase risk of noncompliance due to increasing number of medications to administer to young children.

While the reports described in the literature are encouraging, data from ongoing clinical trials will provide more robust results detailing the efficacy, safety, and optimal dosing of combined 6-MP and allopurinol therapy in children with ALL. Current Children's Oncology Group B-lymphoblastic leukemia protocols (NCT03914625; NCT03959085) include basic recommendations for allopurinol use in 6-MP associated hepatotoxicity as well, thus use of this strategy will likely increase. Overall, the use of allopurinol with reduced dose 6-MP may be a safe and effective intervention in children with ALL who experience 6-MP associated GI toxicity and is reasonable to consider for patients with severe symptoms refractory to other strategies.

## Author Contributions

SEC and RR developed the concept of the paper. SEC wrote the first draft of the paper. SEC, SLC, and RR contributed significant revisions and agreed on the final submitted manuscript. All authors contributed to the article and approved the submitted version.

## Conflict of Interest

The authors declare that the research was conducted in the absence of any commercial or financial relationships that could be construed as a potential conflict of interest.

## References

[1] Hunger SP Mullighan CG. Acute lymphoblastic leukemia in children. N Engl J Med. (2015) 373:1541–52. 10.1056/NEJMra1400972 26465987

[2] Pui CH Evans WE Treatment of acute lymphoblastic leukemia. N Engl J Med. (2006) 354:166–78. 10.1056/NEJMra052603 16407512

[3] Bhatia S Landier W Hageman L Chen Y Kim H Sun CL. Systemic Exposure to thiopurines and risk of relapse in children with acute lymphoblastic leukemia: a Children's Oncology Group study. JAMA Oncol. (2015) 1:287–95. 10.1001/jamaoncol.2015.0245 26181173 PMC4561178

[4] Bostrom B Erdmann G. Cellular pharmacology of 6-mercaptopurine in acute lymphoblastic leukemia. Am J Pediatr Hematol Oncol. (1993) 15:80–6. 10.1097/00043426-199302000-00010 8447563

[5] Melachuri S Gandrud L Bostrom B. The association between fasting hypoglycemia and methylated mercaptopurine metabolites in children with acute lymphoblastic leukemia. Pediatr Blood Cancer. (2014) 61:1003–6. 10.1002/pbc.24928 24415675

[6] Nygaard U Toft N Schmiegelow K. Methylated metabolites of 6-mercaptopurine are associated with hepatotoxicity. Clin Pharmacol Ther. (2004) 75:274–81. 10.1016/j.clpt.2003.12.001 15060506

[7] Dubinsky MC Lamothe S Yang HY Targan SR Sinnett D Theoret Y. Pharmacogenomics and metabolite measurement for 6-mercaptopurine therapy in inflammatory bowel disease. Gastroenterology. (2000) 118:705–13. 10.1016/S0016-5085(00)70140-5 10734022

[8] Friedman AB Brown SJ Bampton P Barclay ML Chung A Macrae FA. Randomised clinical trial: efficacy, safety and dosage of adjunctive allopurinol in azathioprine/mercaptopurine nonresponders (AAA Study). Aliment Pharmacol Ther. (2018) 47:1092–102. 10.1111/apt.14571 29468701

[9] Adam de Beaumais T Fakhoury M Medard Y Azougagh S Zhang D Yakouben K. Determinants of mercaptopurine toxicity in paediatric acute lymphoblastic leukemia maintenance therapy. Br J Clin Pharmacol. (2011) 71:575–84. 10.1111/j.1365-2125.2010.03867.x 21395650 PMC3080646

[10] Cheok MH Evans WE. Acute lymphoblastic leukaemia: a model for the pharmacogenomics of cancer therapy. Nat Rev Cancer. (2006) 6:117–29. 10.1038/nrc1800 16491071

[11] Yang JJ Landier W Yang W Liu C Hageman L Cheng C. Inherited NUDT15 variant is a genetic determinant of mercaptopurine intolerance in children with acute lymphoblastic leukemia. J Clin Oncol. (2015) 33:1235–42. 10.1200/JCO.2014.59.4671 25624441 PMC4375304

[12] Relling MV Schwab M Whirl-Carrillo M Suarez-Kurtz G Pui CH Stein CM. Clinical pharmacogenetics implementation consortium guideline for thiopurine dosing based on TPMT and NUDT15 genotypes: 2018 update. Clin Pharmacol Ther. (2019) 105:1095–105. 10.1002/cpt.1304 30447069 PMC6576267

[13] Mahmoud HH Leverger G Patte C Harvey E Lascombes F. Advances in the management of malignancy-associated hyperuricaemia. Br J Cancer. (1998) 77(Suppl 4):18–20. 10.1038/bjc.1998.432 9647616 PMC2149883

[14] Blaker PA Arenas-Hernandez M Smith MA Shobowale-Bakre EA Fairbanks L Irving PM. Mechanism of allopurinol induced TPMT inhibition. Biochem Pharmacol. (2013) 86:539–47. 10.1016/j.bcp.2013.06.002 23770457

[15] Seinen ML van Asseldonk DP de Boer NK Losekoot N Smid K Mulder CJ. The effect of allopurinol and low-dose thiopurine combination therapy on the activity of three pivotal thiopurine metabolizing enzymes: results from a prospective pharmacological study. J Crohns Colitis. (2013) 7:812–9. 10.1016/j.crohns.2012.12.006 23317929

[16] Lennard L Lilleyman JS. Variable mercaptopurine metabolism and treatment outcome in childhood lymphoblastic leukemia. J Clin Oncol. (1989) 7:1816–23. 10.1200/JCO.1989.7.12.1816 2585022

[17] Lilleyman JS Lennard L. Mercaptopurine metabolism and risk of relapse in childhood lymphoblastic leukaemia. Lancet. (1994) 343:1188–90. 10.1016/S0140-6736(94)92400-7 7909868

[18] Kreijne JE Seinen ML Wilhelm AJ Bouma G Mulder CJ van Bodegraven AA. Routinely established skewed thiopurine metabolism leads to a strikingly high rate of early therapeutic failure in patients with inflammatory bowel disease. Ther Drug Monit. (2015) 37:797–804. 10.1097/FTD.0000000000000213 25853923

[19] Stuckert AJ Schafer ES Bernhardt MB Baxter P Brackett J. Use of allopurinol to reduce hepatotoxicity from 6-mercaptopurine (6-MP) in patients with acute lymphoblastic leukemia (ALL). Leuk Lymphoma. (2020) 61:1246–49. 10.1080/10428194.2019.1702183 31842647

[20] Schmiegelow K Glomstein A Kristinsson J Salmi T Schroder H Bjork O. Impact of morning versus evening schedule for oral methotrexate and 6-mercaptopurine on relapse risk for children with acute lymphoblastic leukemia. Nordic Society for Pediatric Hematology and Oncology (NOPHO). J Pediatr Hematol Oncol. (1997) 19:102–9. 10.1097/00043426-199703000-00002 9149738

[21] Rivard GE Infante-Rivard C Hoyoux C Champagne J. Maintenance chemotherapy for childhood acute lymphoblastic leukaemia: better in the evening. Lancet. (1985) 2:1264–6. 10.1016/S0140-6736(85)91551-X 2866334

[22] Clemmensen KK Christensen RH Shabaneh DN Harila-Saari A Heyman M Jonsson OG. The circadian schedule for childhood acute lymphoblastic leukemia maintenance therapy does not influence event-free survival in the NOPHO ALL92 protocol. Pediatr Blood Cancer. (2014) 61:653–8. 10.1002/pbc.24867 24265159

[23] Landier W Hageman L Chen Y Kornegay N Evans WE Bostrom BC. Mercaptopurine ingestion habits, red cell thioguanine nucleotide levels, and relapse risk in children with acute lymphoblastic leukemia: a report from the Children's Oncology Group study AALL03N1. J Clin Oncol. (2017) 35:1730–6. 10.1200/JCO.2016.71.7579 28339328 PMC5455766

[24] Bell BA Brockway GN Shuster JJ Erdmann G Sterikoff S Bostrom B. A comparison of red blood cell thiopurine metabolites in children with acute lymphoblastic leukemia who received oral mercaptopurine twice daily or once daily: a Pediatric Oncology Group study (now The Children's Oncology Group). Pediatr Blood Cancer. (2004) 43:105–9. 10.1002/pbc.20089 15236274

[25] Cho EM Moon JE Lee SJ Ko CW Severe recurrent nocturnal hypoglycemia during chemotherapy with 6-mercaptopurine in a child with acute lymphoblastic leukemia. Ann Pediatr Endocrinol Metab. (2018) 23:226–8. 10.6065/apem.2018.23.4.226 30599485 PMC6312912

[26] El-Bitar MK Muwakkit SA Dabbagh O. Severe hypoglycemic seizures in a child receiving 6-mercaptopurine. J Pediatr Hematol Oncol. (2011) 33:e75–6. 10.1097/MPH.0b013e3182025507 21343747

[27] Miller MB Brackett J Schafer ES Rau RE. Prevention of mercaptopurine-induced hypoglycemia using allopurinol to reduce methylated thiopurine metabolites. Pediatr Blood Cancer. (2019) 66:e27577. 10.1002/pbc.27577 30548777

[28] Zhang M Bostrom B. Allopurinol reverses mercaptopurine-induced hypoglycemia in patients with acute lymphoblastic leukemia. F1000Res. (2019) 8:176. 10.12688/f1000research.17760.2 30828444 PMC6392151

[29] Bay A Oner AF Cesur Y Dogan M Etlik O Sanli F. Symptomatic hypoglycemia: an unusual side effect of oral purine analogues for treatment of ALL. Pediatr Blood Cancer. (2006) 47:330–1. 10.1002/pbc.20582 16047348

[30] Giamanco NM Cunningham BS Klein LS Parekh DS Warwick AB Lieuw K. Allopurinol use during maintenance therapy for acute lymphoblastic leukemia avoids mercaptopurine-related hepatotoxicity. J Pediatr Hematol Oncol. (2016) 38:147–51. 10.1097/MPH.0000000000000499 26808368

[31] Zerra P Bergsagel J Keller FG Lew G Pauly M. Maintenance treatment with low-dose mercaptopurine in combination with allopurinol in children with acute lymphoblastic leukemia and mercaptopurine-induced pancreatitis. Pediatr Blood Cancer. (2016) 63:712–5. 10.1002/pbc.25841 26878433

[32] Brackett J Schafer ES Leung DH Bernhardt MB. Use of allopurinol in children with acute lymphoblastic leukemia to reduce skewed thiopurine metabolism. Pediatr Blood Cancer. (2014) 61:1114–7. 10.1002/pbc.24913 24376133

[33] Alhubaishi AA. Pancytopenia and septic infection caused by concurrent use of allopurinol and mercaptopurine: a case report illustrating the importance of clinical pharmacist consultation. Am J Case Rep. (2019) 20:1245–7. 10.12659/AJCR.914166 31439827 PMC6717399

[34] Vasudevan A Beswick L Friedman AB Moltzen A Haridy J Raghunath A. Low-dose thiopurine with allopurinol co-therapy overcomes thiopurine intolerance and allows thiopurine continuation in inflammatory bowel disease. Dig Liver Dis. (2018) 50:682–8. 10.1016/j.dld.2018.02.001 29525182

[35] Serpico MR Maltz R Crandall W Bricker J Dotson JL Kim SC. Thiopurine optimization through combination with allopurinol in children with inflammatory bowel diseases. J Pediatr Gastroenterol Nutr. (2018) 67:341–5. 10.1097/MPG.0000000000001986 29601433

